# Assessment of Postural Control and Gait in Patients with Chronic Stroke After Treadmill Perturbation-Based Training: A Randomized Clinical Trial

**DOI:** 10.3390/jcm14176142

**Published:** 2025-08-30

**Authors:** Kamila Niewolak, Joanna Antkiewicz, Laura Piejko, Grzegorz Sobota, Adam Maszczyk, Agnieszka Nawrat-Szołtysik, Józef Opara, Cezary Kucio, Anna Polak

**Affiliations:** 1 Medical and Rehabilitation Center “Solanki”, 88-100 Inowrocław, Poland; k.niewolak@solanki.pl (K.N.); j.antkiewicz@solanki.pl (J.A.); 2 Clinical Department of Physiotherapy in Psychiatry, Faculty of Physical Therapy, Academy of Physical Education in Katowice, 44-200 Rybnik, Poland; l.piejko@awf.katowice.pl; 3 Institute of Physiotherapy and Health Sciences, Academy of Physical Education, 40-065 Katowice, Poland; a.nawrat-szoltysik@awf.katowice.pl (A.N.-S.); j.opara@awf.katowice.pl (J.O.); c.kucio@awf.katowice.pl (C.K.); 4 Department of Clinical Physiotherapy, Clinical Psychiatric Hospital, 44-200 Rybnik, Poland; 5 Institute of Sport Sciences, Academy of Physical Education, 40-065 Katowice, Poland; g.sobota@awf.katowice.pl (G.S.); a.maszczyk@awf.katowice.pl (A.M.); 6 Department of Physiotherapy in Internal Medicine, Faculty of Physical Therapy, Academy of Physical Education, 40-065 Katowice, Poland; 7 Department of Clinical Physiotherapy, Faculty of Physical Therapy, Academy of Physical Education, 40-065 Katowice, Poland

**Keywords:** stroke, physical therapy, treadmill, balance perturbations, perturbation-based training, postural control, gait quality

## Abstract

**Background:** After ischemic heart disease, stroke is globally the second leading cause of death and the second most common cause of disability. The rehabilitation of patients with chronic stroke increasingly uses advanced technologies, such as treadmill perturbation-based training (TPBT). While the results of studies with TPBT are promising, they are inconclusive due to the limited number of works and inconsistent research methodologies. Therefore, more randomized clinical trials (RCTs) are needed to evaluate TPBT’s efficacy and applicability in post-stroke rehabilitation. This prospective RCT was designed to assess whether and to what extent TPBT can improve postural balance and gait quality and reduce fear of falling in patients with chronic stroke. **Methods:** Fifty individuals who were at least six months post-stroke were enrolled in the trial and randomly assigned to the experimental group (EG; n = 25) to receive the TPBT or the control group (CG; n = 25) to receive overground gait and balance training. Both groups exercised six times per day for three weeks. **Results:** The Berg Balance Scale showed post-intervention that the postural balance improved significantly in both groups (EG, *p* = 0.001 and CG, *p* = 0.009), but the change did not statistically significantly differentiate the EG from the CG (*p* = 0.256). The significant improvements in walking speed over the distance of 10 m (*p* = 0.015) and fear of falling (*p* = 0.002) in the CG were not significantly different from those in the EG (*p* = 0.543). **Conclusions:** TPBT applied to patients with chronic stroke improves their postural control comparably to conventional gait and balance training but does not enhance their gait quality.

## 1. Introduction

Stroke is the second most common cause of death after ischemic heart disease and the second leading cause of disability globally [1]. The Global Burden of Disease Study [1] estimated that, in 2016, 80.1 million people were living with stroke worldwide, of whom 13.7 million suffered a first-ever stroke. Stroke, mostly of the ischemic type (84%), was diagnosed in 41.1 million women and 39.0 million men. According to the study’s stroke mortality statistics, 5.5 million people died of a stroke in 2016, and 116.4 million became disabled as a result of acute or chronic stroke [1]. Between 1990 and 2013, the global number of strokes and stroke-related deaths and disabilities among individuals aged 20–64 years of age increased significantly by 1.4–1.8 times for ischemic strokes and 1.2–1.9 times for hemorrhagic strokes [2].

After a systematic review of clinical trials, Steward et al. [3] pointed to physical activity as a vital element of stroke prevention and treatment. Physical exercise reduces the effects of neurological damage and stimulates brain plasticity in stroke survivors, with the greatest improvement in motor function occurring in the early post-stroke phase. In the chronic phase (six months after a stroke and later), many patients reach a plateau or experience slower motor recovery, which dispirits them and causes them to withdraw from physiotherapy [4,5]. Studies show, however, that regular physical exercise can offer significant health improvements even months after stroke [6,7,8,9,10,11,12], as well as in patients who have reached a recovery plateau [6]. This means that patients in the late post-stroke period should not be excluded from training programs based on their inclusion of physical exercises that require learning new tasks, engaging conscious attention, and stimulating cognitive functions [13].

The most common consequences of stroke are postural balance, control, and gait impairments. France by De Peretti et al. [14] reported balance disorders in 50% of patients who are post-stroke. Three months after experiencing a stroke, gait disorders still affect 60% of patients, with 20% needing wheelchairs to move around [15,16,17]. The distance that an adult who suffered an acute or subacute stroke can cover is 40–50% shorter than that which a healthy adult can walk [18]. De Haart et al. [19] found abnormal postural control to be one of the main causes of post-stroke mobility impairment. Pohl et al. [18] concluded that balance improvement was the strongest predictor of the gait distance that patients could walk three months after stroke.

Patients who are post-stroke show many changes in their motor strategies for postural control, among which body weight asymmetry, delayed and reduced anticipatory postural adjustments, synergistic muscle coactivation, and abnormal postural tilt are the most common. The majority of these changes are related to the impaired central nervous system, but some appear to be adaptive compartments [20]. Lamb et al. [21] concluded that postural and balance impairments are the strongest predictors of falls in stroke survivors, and Belgen et al. [22] reported that individuals who had experienced falls were more fearful of falling in the chronic post-stroke phase and showed lower falls-related self-efficacy (*p* = 0.04) and more depressive symptoms (*p* = 0.02) than those who had not fallen. Evidence has also been presented that individuals with a history of multiple falls have poorer balance (*p* = 0.02), greater fear of falling, and use more medications (*p* = 0.04) than non- or one-time fallers [22]. Falls are a major cause of injuries leading to disability, social isolation, and reduced quality of life [23]. People who fear falling tend to limit their daily activity and adopt a sedentary lifestyle, which further increases their risk of falling, dependence on others, and disability [24].

For the above reasons, exercises that improve balance and gait quality play a significant role in stroke rehabilitation [13]. To evaluate the ability of different types of exercise to improve balance in chronic stroke survivors, Van Duijnhoven et al. [25] conducted a systematic review and meta-analysis of 43 randomized clinical trials (RCTs) of moderate or high methodological quality (PEDro score ≥ 4) that involved a total of 1613 patients. The authors of most of the studies (n = 28) assessed the participants’ postural balance using the Berg Balance Scale (BBS). Subgroup analyses of studies that reported BBS outcomes showed that only balance- and weight-shifting training and gait training had a significant and positive effect on postural balance.

Programs to rehabilitate the balance and gait quality in chronic stroke survivors use various approaches and utilize modern technologies, including treadmill training [10,26,27,28,29,30,31]. Treadmill exercises, especially those performed without body weight support (BWS) [10,31], are significantly more effective in improving gait speed [10,31] and step length and width [31] than overground training.

Perturbation-based balance training (PBT), which has, in recent years, been introduced as a therapy for balance and gait impairments of various origins, is usually performed using equipment that induces changes in ground surface compliance [32,33,34] or moveable floor platforms [35,36,37,38,39,40]. Also used are treadmills [30,41,42,43,44,45,46,47], where balance disturbances are induced by two independently moving belts [48], belt accelerations or decelerations [30,41,43,45,46], and forward–backward as well as lateral belt translations [44]. A systematic review of eight RCTs by Mansfield et al. [49] found patients who completed the PBT to have a lower risk of falling (*p* = 0.02) and to report fewer falls than controls who did not perform PBT (*p* = 0.007). The RCTs involved 404 adults, including healthy and frail older adults (>60 years) and adults with Parkinson’s disease.

To the authors’ knowledge, there are only four RCTs [40,44,45,50] in which patients with chronic stroke performed PBT. In two of them, authored by Esmaeili et al. [45] and Hu et al. [44], the participants had 9 [45] and 20 [44] training sessions spanning 3 [45] and 4 [44] weeks, respectively. In Esmaeili et al. [45], balance perturbations were induced by accelerating and decelerating the treadmill belt, while Hu et al. [44] also used lateral belt translations. The studies produced inconsistent results. Esmaeili et al. [45] reported that PBT improved participants’ dynamic balance but not their gait speed, whereas Hu et al. [44] observed improvement in gait speed but found none in dynamic balance. The authors of the other two RCTs [40,50] used moveable floor platforms [50] and applied changes in ground surface compliance [40]. Based on the outcomes of a single PBT session, the authors of one of the studies [40] concluded that PBT offered protection from falling by helping develop adaptive responses. The second study was a pilot RCT with only 12 patients, who were equally divided between two groups. After 20 PBT sessions were performed over 4 weeks, its authors observed improvement in participants’ dynamic balance and gait speed. Three clinical studies [46,47,51] where patients with chronic stroke performed PBT did not have control groups. The studies only recruited 10 [51] and 12 [46,47] patients, who performed 3 [46], 10 [51], and 15 [47] training sessions, respectively. All three studies reported improvements in participants’ gait speed [47,51] and gait quality [46,51].

The cited studies indicate that PBT, including that which involves the use of a treadmill, is capable of improving the balance and gait quality in patients with chronic stroke. However, given that RCTs of this type are few and their results are inconsistent due to the variety of training methods used by their authors, more RCTs are needed to ascertain the most appropriate training methods and their ability to influence the balance and gait quality in patients with chronic stroke.

This RCT was designed to determine whether, and to what extent, treadmill perturbation-based balance training (TPBT) improves the balance and gait quality and reduces fear of falling in patients with chronic stroke. It also sought to establish whether the effect of TPBT on these parameters would be comparable to or greater than that of overground gait and balance training.

## 2. Materials and Methods

**Trial design:** This prospective, randomized, controlled clinical trial was designed to compare body balance, gait quality, and fear of falling in patients with chronic stroke divided into two parallel groups that participated in conventional stroke rehabilitation enhanced by treadmill perturbation-based training (TPBT; the experimental group, EG) or conventional stroke rehabilitation supplemented by traditional overground gait and balance training (the control group, CG). The trial adhered to the principles of the Declaration of Helsinki and was approved by the Bioethics Committee for Scientific Research at the Jerzy Kukuczka Academy of Physical Education in Katowice, Poland on 9 July 2020 (Resolution No. 5/2020). It was prospectively registered with the International Standard Randomized Controlled Trial Number Registry: ISRCTN17138124.

**Setting and participants.** All participants were under the care of the same single medical and rehabilitation center. They were recruited for this study based on the following eligibility criteria: age ≥ 18 years; first-ever ischemic or hemorrhagic stroke occurring ≥6 months prior; independent ambulation over 10 m (assistive devices such as a cane or walker permitted); minimum gait speed of 0.4 km/h; spasticity of the affected lower limb graded between 0 and 2 on the Modified Ashworth Spasticity Scale; Brunnström Recovery Scale stages III–VI for the lower limb; and written informed consent to participate. 

Excluded from the trial were patients who suffered more than one stroke; with contraindications to physical exercise or subarachnoid hemorrhage; unable to comprehend verbal instructions; with a Mini-Mental State Examination (MMSE) score ≤ 24 and a Geriatric Depression Scale (GDS) score ≥ 20; affected by severe aphasia impeding communication (assessed by a speech therapist); with hemianopsia or hemineglect; with stroke onset < 6 months prior; with complete hip joint ankylosis; with lower limb length discrepancy exceeding 3 cm; presenting with cardiopulmonary insufficiency precluding walking more than 10 m or conditions affecting gait and balance other than those related to stroke (central or peripheral nervous system disorders, fractures of the spine or limbs within the past year, osteoporosis, acute inflammatory diseases of internal organs or the musculoskeletal system, limb amputations, vision impairments, malignancies under treatment, post-chemotherapy neuropathies, diabetic neuropathy, etc.).

All participants completed outpatient and inpatient rehabilitation programs prior to enrollment in the trial, which was part of a broader rehabilitation plan. Their demographic data were collected through standard interviews, physical examinations, and a review of their medical records.

**Randomization:** A person unrelated to the trial received 50 slips of paper, 25 of which were marked with “A” (CG) and the other 25 of which were marked with “B” (EG), and placed each slip in one of 50 envelopes according to a randomized computer-generated sequence. After sealing, the envelopes were delivered to the research manager, who opened them in the presence of a physiotherapist to randomly assign willing and consenting patients (or those who had consenting legal guardians) to either group.

**Blinding:** Both the physiotherapist who performed initial and final clinical assessments of the participants and the statistician in charge of data analysis were blinded to group assignments.

**Intervention: post-stroke therapy administered to both groups:** All patients participated in a 3-week rehabilitation program based on conventional post-stroke therapy. Sessions of 2.5 h, designed according to the best clinical practices, were delivered Monday through Saturday. The exercises they included aimed to improve participants’ movement patterns and normalize their muscle tone.

**Treadmill training in the EG.** In addition to receiving conventional therapy, participants in the EG exercised on a treadmill (Balance Tutor; MediTouch, Ltd., Tel Aviv, Israel) controlled by software capable of inducing postural perturbations in the frontal and sagittal planes during walking. A harness connected to an overhead support system protected exercising patients from falling without supporting their body weight. Handrail use was prohibited. Sessions were performed daily for 3 weeks, Monday through Saturday. The treadmill speed was always set according to patient preference and recorded. Exercise duration and difficulty were progressively increased (Table 1).

**Conventional gait and balance training in the CG:** The purpose of this training was to improve participants’ performance of functional tasks, such as standing under various conditions (with reduced sensory input, narrowed base of support, while reaching for and catching objects, responding to unexpected manual perturbations), sit-to-stand transfers, multidirectional stepping, overground walking, and walking up and down stairs. Emphasis was placed on stability and normal weight-bearing patterns during training tasks. The use of assistive devices was permitted if needed. Participants were encouraged to use postural control and movement strategies they developed in daily living. The length of the sessions was progressively increased to match the duration of the sessions in the EG: 10 min on days 1–3, 15 min on days 4–6, and 20 min between day 7 and day 18.

**Measures:** Clinical assessments of participants were performed twice, on the day preceding the 3-week rehabilitation program and on the first day after it ended. Their cognitive function was determined pre-intervention using the MMSE [52], and depressive symptoms were assessed with the GDS [53]. Motor function and the stages of lower limb recovery were measured using the Brunnström Recovery Scale [19,54], spasticity severity was evaluated with the Modified Ashworth Scale [55,56], and independence in activities of daily living was established using the 100-point Barthel Index [57].

Participants’ static and dynamic body balance, gait quality, and fear of falling were assessed pre- and post-intervention. Balance measurements were performed using functional tests: the BBS for static and dynamic balance [58], the Functional Reach Test (FRT) for dynamic balance [59], and the Timed Up and Go (TUG) Test for dynamic balance [60]. The BBS is a 14-item objective tool with excellent interrater reliability (ICC = 0.97) and intrarater reliability (ICC = 0.98) for patients with chronic stroke [61,62]. It also has an adequate ability to predict fall occurrence (area under the curve (AUC) (95% CI) = 0.813 [0.691–0.936], sensitivity = 75%, and specificity = 76.9%) [63]. The FRT assesses dynamic balance in simple tasks and has intersubject reliability of 0.987 (0.983–0.992) and intrasubject reliability of 0.983 (0.979–0.989) for patients with stroke [64]. The TUG Test that evaluates mobility, balance, walking ability, and fall risk in older adults has excellent test-retest reliability (ICC = 0.96) for patients with chronic stroke [65]. Its results show a high correlation (r = 0.86–0.92) with the results of other mobility, balance, exercise tolerance, and fall risk tests, such as the Comfortable Gait Speed Test, Fast Gait Speed Test, Stair Climbing Test, and 6-Minute Walk Test [65].

Participants’ static balance was additionally assessed using a stabilometric platform (Zebris FDM-T; Reha-walk, MaxxusDaum, Allgäu, Germany), which recorded forces and torques at a sampling rate of 80 Hz. During measurements, participants stood quietly for 60 s with their eyes open, feet shoulder-width apart, and arms at their sides. Gait speed was assessed with the 10-Meter Walk Test (10MWT) [66], which measures walking speed over a short distance in meters per second. The test has excellent test-retest reliability ((ICC) = 0.95–0.99) [67] for patients with chronic stroke, as well as excellent reliability for both comfortable (ICC = 0.94) and fast (ICC = 0.97) gait speeds [65]. The 10MWT has also been demonstrated to have high predictive validity and excellent correlation with dependence in instrumental activities of daily living (r = 0.76) and the Barthel Index (r = 0.78) [68]. Spatiotemporal gait parameters were also evaluated on a treadmill (Zebris FDM-T; Reha-walk, MaxxusDaum, Allgäu, Germany). For patients to be able to find a comfortable walking speed, they walked on the treadmill to try it before the test; during this trial, the belt speed was decreased and increased twice. A 30 s measurement was taken and its result was included in the analysis.

Participants’ fear of falling was assessed using the 16-item Falls Efficacy Scale–International (FES-I) [69,70], which showed excellent internal consistency (cronbach’s α = 0.96) and test–retest reliability (ICC = 0.96) in a group of 704 seniors aged 60–95 years who were at risk of falls. Its high validity and reliability have also been confirmed by a study on individuals with neurological disorders, including vestibular disorders (ICC = 0.94) [71] and Parkinson’s disease (ICC = 0.91 to 0.94) [72].

**Outcomes: primary outcomes:** Primary outcomes of the trial included static and dynamic body balance and gait speed, which were measured at baseline and after 3 weeks of the rehabilitation program using the BBS and the 10MWT, respectively.

**Secondary outcomes**: Secondary outcomes included dynamic body balance assessed by the TUG Test and the FRT; static body balance assessed on a stabilometric platform (Zebris FDM-T; Reha-walk, MaxxusDaum, Allgäu, Germany); gait quality assessed by a walking test on a treadmill (Zebris FDM-T; Reha-walk, MaxxusDaum, Allgäu, Germany); and fear of falling assessed using the FES-I questionnaire. These tests were also conducted at baseline and after 3 weeks of rehabilitation.

**Statistical analysis**: **sample size calculation**: To determine the appropriate sample size for the EG and CG, a pilot study was conducted with 12 patients with chronic post-stroke, randomized to receive either perturbation-based treadmill training (EG) or conventional overground gait and balance training (CG) according to clinical guidelines. Body balance and gait speed over 10 m were measured after three weeks of intervention using the BBS and 10MWT, respectively. Given the unimodal distribution of scores and skewness and kurtosis values below 2.5, the arithmetic mean and standard deviation were used to describe central tendency and dispersion. For the sample size calculation, the significance level (type I error) was set at α = 0.05 and the type II error at β = 0.1 (power = 0.90). A minimum clinically important difference (MCID) of 25% between pre- and post-intervention values was assumed. Based on these outcomes and Student’s *t*-distribution, each group required at least 23 participants. To account for potential dropouts, two additional participants per group were recruited, resulting in a final sample size of 25 per group.

**Intention-to-treat analysis**: All 50 randomized patients were included in the intention-to-treat (ITT) analysis. For participants who did not complete the intervention, missing data were imputed using linear regression based on prior assessments and clinical similarity, according to the formula *y* = *a*·*x* + *b*, where *a* is the regression coefficient and *b* is the intercept. This approach assumed a linear trend in the variables under study. The impact of imputation was evaluated by sensitivity analysis comparing ITT and per-protocol outcomes; the absence of statistically significant differences indicated that imputation did not materially affect the trial’s results.

**Statistical analysis**. All statistical analyses were performed using Statistica software (version 15, StatSoft Polska Sp. z o.o., Kraków, Poland). A significance level of *p* ≤ 0.05 was adopted for all tests. The normality of pre-intervention variable distributions was assessed using the Shapiro–Wilk test, and the homogeneity of variances was evaluated with Levene’s test. As the data did not meet assumptions of normality and homogeneity of variance, non-parametric tests were used throughout the analysis. For descriptive statistics, both means with standard deviations and medians with interquartile ranges were reported, reflecting the unimodal distributions and skewness/kurtosis values below 2.5. Baseline characteristics were compared between groups using the Mann–Whitney U test for continuous variables and the chi-square test for categorical variables. Within-group pre- and post-intervention results were compared using the Wilcoxon signed-rank test. Between-group comparisons of outcome changes were performed using the Mann–Whitney U test.

Distributions violated normality and homoscedasticity; therefore, we used non-parametric tests (Wilcoxon within-group; Mann–Whitney between-group changes) under an intention-to-treat framework. Given baseline balance, this approach yields inferences comparable to baseline-adjusted models. Per reviewer request, we additionally performed a rank-based ANCOVA (Quade/Conover–Iman) as a sensitivity analysis, specifying post-intervention scores as the dependent variable, the baseline score as covariate, and group as fixed factor.

For all primary and secondary outcomes, effect sizes were estimated using Cohen’s d, calculated from group means and pooled standard deviations. This approach provides a standardized measure of the magnitude and precision of observed effects, facilitating interpretation and comparison across outcomes and studies. All analyses adhered to the intention-to-treat principle, and sensitivity analyses were conducted to confirm the robustness of the findings. All 50 randomized participants were included in the intention-to-treat analysis. Sensitivity analysis comparing ITT and per-protocol populations revealed no significant differences in the main outcomes.

## 3. Results

**Figure 1:**
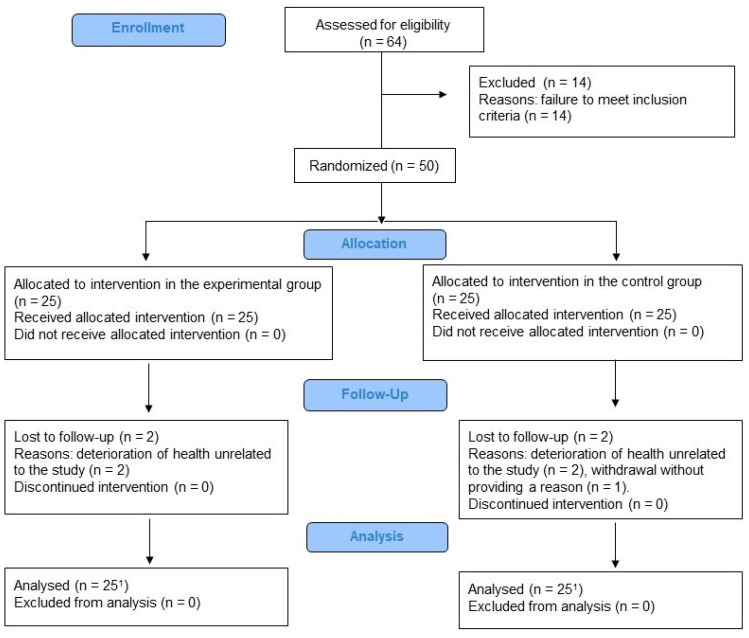
Diagram flow of this study (^1^ in both the baseline and final outcome analyses, all randomized patients were included in accordance with the intention-to-treat analysis).

Between 28 March 2022, and 30 June 2022, 64 patients were screened for this trial. Fifty patients who met the inclusion criteria were randomly assigned to either the EG or CG. Five patients (10%) did not complete the trial. Two patients in the EG and two in the CG withdrew due to a deterioration in health unrelated to the procedures used in this study, while one patient in the CG withdrew without providing a reason. The remaining 45 participants completed the intervention. The participant flow is presented in Figure 1.

### 3.1. Baseline Characteristics

The participants’ ages ranged from 45 to 82 years. The BMI distribution was as follows: 15 participants (30%) had normal weight (BMI 18.5–24.99 kg/m^2^), 27 (54%) were overweight (BMI 25.0–29.99 kg/m^2^), 4 (8%) had obesity class I (BMI 30.0–34.99 kg/m^2^), and 4 (8%) had obesity class II (BMI 35.0–39.99 kg/m^2^). All participants (100%) had suffered an ischemic stroke 6–28 months prior to their enrollment. Twenty-four participants (48%) had right-sided paresis, and 26 (52%) had left-sided paresis. In 24 patients (48%), the dominant limb was affected; in 26 (52%), the non-dominant limb. The Brunnstrom Recovery Stages for the lower limb were as follows: stage 3 in 4 (8%), stage 4 in 6 (12%), stage 5 in 8 (16%), and stage 6 in 32 (64%) participants. Increased muscle tone was not observed in 9 (58%) participants (grade 0, Modified Ashworth Scale), it was slight in 9 (18%, grade I), and it was moderate in 3 (6%, grade II). Apart from one participant (2%) with severe dependence in activities of daily living (Barthel Index 21–60), all others (98%) were moderately dependent (Barthel Index 61–90). At baseline, there were no statistically significant differences between the EG and CG for any demographic or clinical variables (Table 2 and Table 3).

### 3.2. Primary Study Outcomes

**BBS:** After the intervention, both groups improved significantly on the BBS. In the EG, the mean BBS score increased from 43.83 (SD 8.85) to 49.50 (SD 7.82) (within-group *p* = 0.001). In the CG, the mean increased from 46.00 (SD 9.80) to 49.23 (SD 9.80) (within-group *p* = 0.009). The between-group difference in BBS change was 2.44 points (CI: −8.93 to 13.81), with a Cohen’s d of 0.27 (CI: −0.99 to 1.52), which indicates a small effect size and no statistically significant difference between groups (pre-intervention *p* = 0.408, post-intervention *p* = 0.256; Table 4).

**10MWT:** The gait speed improved significantly only in the CG, increasing from 0.63 m/s (SD 0.25) to 0.70 m/s (SD 0.21) (within-group *p* = 0.015). The between-group difference in change was −0.07 m/s (CI: −0.37 to 0.23) and Cohen’s d = −0.30 (CI: −1.55 to 0.96), which, again, indicates a small and non-significant effect (pre-intervention *p* = 0.948, post-intervention *p* = 0.543; Table 4).

### 3.3. Secondary Study Outcomes

**FRT:** The functional reach increased significantly post-intervention only in the CG (from 29.87 (SD 9.29) cm to 34.50 (SD 8.64) cm, *p* = 0.021). The between-group difference in change was 4.63 cm (99.99% CI: −5.24 to 14.50) and Cohen’s d = 0.52 (CI: −0.60 to 1.63); however, this difference was not statistically significant (pre *p* = 0.150, post *p* = 0.870; Table 4).

**TUG test:** The CG required significantly less time post-intervention (from 13.07 (SD 6.56) to 11.13 (SD 4.88) seconds, *p* = 0.009). The between-group difference in change was −1.94 s (CI: −8.30 to 4.42) and Cohen’s d = −0.34 (CI: −1.44 to 0.77), with no significant difference between groups (pre *p* = 0.958, post *p* = 0.623; Table 4).

**FES-I:** A significant decrease in fear of falling was observed only in the CG (from 28.47 (SD 8.96) to 26.10 (SD 9.10), *p* = 0.002). The between-group difference in change was −2.37 points (CI: −12.31 to 7.57) and Cohen’s d = −0.26 (CI: −1.37 to 0.84); the differences between groups were not significant (pre *p* = 0.525, post *p* = 0.527; Table 4).

**Static balance (stabilometric platform)**: No statistically significant changes were observed in the CoP path length or 95% confidence ellipse area in either group (EG: *p* = 0.385 and *p* = 0.367; CG: *p* = 0.824 and *p* = 0.741). The between-group differences in change were 0.00 (CI: −11.01 to 11.01) and Cohen’s d = 0.00 (CI: −1.10 to 1.10), with no significant differences at baseline or post-intervention (CoP path length: *p* = 0.678 and *p* = 0.675; ellipse area: *p* = 0.597 and *p* = 0.672; Table 5).

**Spatiotemporal gait parameters:** Significant changes post-rehabilitation were observed only in the EG, where the step length increased for both legs (right: from 27.4 (SD 8.2) cm to 30.3 (SD 9.0) cm, *p* = 0.015; left: from 27.5 (SD 8.9) cm to 30.1 (SD 9.8) cm, *p* = 0.449). The between-group differences in step length change were 2.90 cm (right leg: CI: −6.12 to 11.92) and Cohen’s d = 0.35 (CI: −0.76 to 1.46), with no significant differences between groups (right: *p* = 0.932; left: *p* = 0.750). In the EG, the cadence decreased significantly (from 82.4 (SD 14.7) to 79.6 (SD 15.9) steps/min, *p* = 0.029), but the between-group difference in change was not significant (*p* = 0.250). Other gait parameters showed no significant within- or between-group differences (Table 6).

## 4. Discussion

The BBS scores showed that both treadmill perturbation-based training performed six days a week for three weeks (the EG) and overground gait and balance training of matching frequency and duration (the CG) significantly improved the body balance in patients with chronic stroke. The post-intervention outcomes of the 10MWT demonstrated that the overground walking speed significantly improved only in the CG, but the change was not statistically significantly bigger than that of the EG. Spatiotemporal gait measurements obtained for participants walking on the treadmill indicated a statistically significant increase in step length (cm) and a statistically significant decrease in cadence (steps/min) only in the EG. Even so, the spatiotemporal gait parameters in the EG were not significantly better post-intervention than in the CG. These results are inconclusive as to whether three weeks of treadmill perturbation-based training and conventional overground gait training of the same duration can improve gait efficiency in patients with chronic stroke.

**Comparison of this study’s results with other studies:** the authors’ knowledge, there are only two RCTs [44,45] that are comparable to this study because both of them used treadmill perturbation-based training and patients with chronic stroke. However, they are also different from our trial in some respects. Esmaeili et al. [45] conducted a pilot RCT involving 18 participants (EG: n = 10, CG: n = 8). The EG performed treadmill training with balance perturbations induced by speed changes, while the CG trained on the treadmill without perturbations. The exercises lasted up to 30 min and included a total of nine training sessions over 3 weeks. The study showed a significant improvement in dynamic balance in the EG compared with the CG (*p* = 0.007), whereas the gait speed did not differ significantly between the groups. These findings confirm the observations of our study that TPBT may improve the dynamic balance in patients with chronic stroke, without significantly affecting gait speed. Hu et al. [44] conducted an RCT with 40 patients (EG: n = 20, CG: n = 20), who trained daily for 30 min, five days a week, over four weeks (20 sessions in total). The EG performed TPBT with a gradually increasing amplitude, speed, and acceleration of perturbations, while the CG participated in conventional gait and balance training. After 2 and 4 weeks, significant improvements in both the normal and fast walking speed were observed in the EG, whereas the CG did not show such changes. The dynamic balance improved in the EG, but without significant between-group differences. Unlike in our study, Hu et al. [44] observed improvements in walking speed but not balance. The time since stroke, degree of lower limb recovery, and TPBT methodology were comparable to our study, but differences in patient age may explain the discrepancies—the patients in Hu et al.’s [44] trial were younger than in our study, with a mean age of 48.7 ± 14.8 years in the EG and 43.1 ± 16.2 years in the CG.

The effects of TPBT on gait quality have also been analyzed in three clinical trials without control groups [46,47,51]. The results of these studies showed that TPBT may improve the gait quality in patients with chronic stroke, which was not observed in our study. TPBT was also found to induce beneficial compensatory reactions of the trunk [46] and lower limbs [46], protecting against falls, which were not assessed in our trial. 

Dusane et al. [46] demonstrated that different forms of TPBT (slips, trips, mixed exercises) have distinct effects on the gait parameters and compensatory trunk movements in stroke patients. Slip training improved stability by increasing the step length and reducing the number of compensatory steps (*p* < 0.05), trip training reduced the step length, trunk inclination angle, and compensatory steps (*p* < 0.05), and mixed training reduced both the step length and trunk inclination (*p* < 0.05). These findings confirm that TPBT can support compensatory mechanisms and reduce fall risk, but they cannot be directly compared with our study, which did not specifically analyze the effects of different perturbation modalities on gait.

Osman et al. [47] reported significant improvements in walking speed at both the preferred (*p* = 0.003) and maximal pace (*p* = 0.010), as well as a reduction in the number of treadmill falls (*p* = 0.015). The authors applied 15 training sessions over 6 weeks. In our study, 18 sessions were condensed into 3 weeks, which may have limited the gait quality improvement. It is also worth noting that, in Osman et al. [47], perturbations were applied randomly but always during the stance phase of the non-paretic leg, which was not consistently followed in our study.

Punt et al. [51] included patients who were ≥12 months post-stroke with a history of falls. The intervention consisted of 10 training sessions over 6 weeks, lasting 30–60 min depending on participants’ abilities. Perturbations were induced by accelerating, decelerating, and laterally tilting the treadmill belt. Upon completion, significant improvements were observed in walking speed, step length, and reductions in step time and swing phase duration, which indicated an overall improvement in gait quality. It is possible that post-stroke patients with a history of falls are more responsive to interventions that enhance protective mechanisms against balance loss. The literature highlights that such individuals typically demonstrate poorer postural and motor control as well as greater fear of falling, which increases the risk of recurrent incidents [14,15,16,17,21,22]. At the same time, numerous studies confirm that targeted balance and gait training in the chronic stroke phase can significantly improve stability and reduce fall risk [73,74]. This may explain the observed improvement in gait quality after TPBT in patients with a history of falls, in contrast to our study, where this inclusion criterion was not considered.

In summary, although our findings and the available literature [44,45,46,47,51] are not entirely consistent, they confirm the potential of TPBT in improving the dynamic balance [45,47] and gait quality [44,46,47,51] in patients with chronic stroke. These findings are promising; however, due to the limited number of RCTs with control groups and methodological heterogeneity, further studies are needed to clearly identify the most effective types of TPBT for improving balance and gait quality and reducing fear of falling in patients with chronic stroke. It is important to conduct studies that allow tailoring TPBT methods to individual patient needs.

**Digital technologies and machine learning in the assessment of body balance, gait, and fall risk:** In recent years, technologies supporting post-stroke rehabilitation, including wearable systems, virtual reality, and telemedicine, have been rapidly developing [75]. They enable objective assessment of gait and balance as well as therapy personalization. Abdollahi et al. [76] showed that motion tests (TUG, 10MWT) and postural sway analysis predict fall risk better than subjective scales. In more recent studies, the same authors [77] applied machine learning algorithms (random forest), achieving high accuracy (91%) in classifying fall risk using a single inertial sensor. The integration of TPBT with objective measurements and AI-supported analysis may represent a novel strategy to improve patient safety. Our results suggest that TPBT reduces fall risk factors that are detected both by traditional tests and modern digital tools. Future studies should incorporate sensor-based technologies and machine learning models to monitor the effectiveness of TPBT and predict individual fall risk, which would pave the way for more personalized and preventive rehabilitation.

**Strengths of this study:** This study was designed as a randomized controlled trial with a control group. The sizes of the EG and the CG were determined based on the results of a pilot study. All training sessions were provided at the same rehabilitation center and were supervised by physiotherapists to ensure that both groups exercised in a uniform and reliable manner. All assessments were carried out by the same team of physiotherapists. The results for four participants who failed to complete the trial were approximated and processed statistically using intention-to-treat (ITT) analysis.

**Limitations of this study:** One limitation of this trial is that the intervention results were only compared with the baseline values of the parameters under study, without considering TPBT’s long-term impacts. Unfortunately, a follow-up assessment could not be performed because most of the trial participants lived in locations that were distant from the rehabilitation center. Because of the nature of the intervention, blinding of the participants and medical personnel was not possible, either. As a robustness check, we provide a rank-ANCOVA (Quade/Conover–Iman) in Supplementary Table S1; this aligns with guidance favoring the use of ANCOVA on post-treatment values adjusted for baseline.

## 5. Conclusions

This trial demonstrated that 18 sessions of treadmill perturbation-based training performed by patients with chronic stroke over 3 weeks positively influenced their body balance, comparably to conventional overground gait and balance training. However, the PBT neither reduced participants’ fear of falling nor increased their walking speed. In the EG, spatiotemporal gait analysis revealed a significant increase in step length (cm) and a significant decrease in cadence (steps/min). These changes do not conclusively indicate whether three weeks of the PBT can improve the gait efficiency in patients with chronic stroke. More RCTs are needed to create PBT protocols that will improve balance and gait and reduce the fear of falling in these patients the most effectively

## Figures and Tables

**Table 1:** Perturbation-based treadmill training protocol.

Exercise	Total Exercise Duration	Walking Forward Without Perturbations	Lateral Translations				Forward-Backward Translations		
			Duration	Frequency	Amplitude	Direction	Duration	Frequency	Amplitude
1	10 min	7 min	3 min	Every 30 s	7 cm	Only toward the paralyzed side			
2					8 cm				
3					10 cm				
4–6	15 min	0 min	5 min	Every 30 s	15 cm	Toward the paralyzed and unaffected sides	10 min	Every 30 s	10 cm
7–12	20 min		7 min	Every 30 s	18 cm	Toward the paralyzed and unaffected sides	13 min	Every 30 s	15 cm
13–18	20 min		7 min	Every 30 s	20 cm	Toward the paralyzed and unaffected sides	13 min	Every 30 s	15 cm

**Table 2:** Pre-intervention characteristics of the groups.

Characteristics	Experimental Group (n = 25)	Control Group (n = 25)
^1^ Gender: female/male [n (%)]	10 (40%)/15 (60%)	9 (36%)/16 (64%)
^2^ Age [years]:		
Mean (SD)	60.87 (11.01)	64.20 (6.38)
Median (Q1, Q3)	62.0 (54.6, 69.4)	65.0 (60.7, 67.3)
^1^ BMI [no. of Pts (%)]:		
<18.5 (underweight)	0 (0%)	0 (0%)
18.5–24.99 (normal)	8 (32%	7 (28%)
25.0–29.99 (overweight)	13 (52%)	14 (56%)
30.0–34.99 (class I obesity)	2 (8%)	2 (8%)
35.00–39.99 (class II obesity)	2 (8%)	2 (8%)
^1^ Ischemic stroke/hemorrhagic stroke [no. of Pts (%)]:	25 (100%)/0 (0%)	25 (100%)/0 (0%)
^2^ Time since stroke [months]:		
Mean (SD)	12.33 (6.56)	11.83 (5.95)
Median (Q1, Q3)	12.0 (7.6, 16.4)	12.0 (7.0, 15.0)
^1^ Affected side: right/left	11 (44%)/14 (56%)	13 (52%)/12 (48%)
^1^ Affected side [dominant/non-dominant] [no. of Pts (%)]	10 (40%)/15 (60%)	14 (56%)/11 (44%)
^1^ Brunnström Recovery Scale [number of Pts (%)]		
Stage III	1 (4%)	3 (12%)
Stage IV	4 (16%)	2 (8%)
Stage V	5 (20%)	3 (12%)
Stage VI	15 (60%)	17 (68%)
^1^ Modified Ashworth Spasticity Scale: 0/I/II [number of Pts (%)]		
Grade 0	18 (72%)	21 (84%)
Grade 1	5 (20%)	3 (12%)
Grade 2	2 (8%)	1 (4%)
^1^ Barthel Scale [points]:		
Mean (SD)	94.5 (9.5)	95.83 (6.83)
Median (Q1, Q3)	95.0 (88.0, 100.0)	97.0 (91.0, 100.0)
0–20 points (total dependence)	0 (0%)	0 (0%)
21–60 points (severe dependence)	1 (4%)	0 (0%)
61–90 points (moderate dependence)	24 (96%)	25 (100%)
^2^ Berg Balance Scale [points]:		
Mean (SD)	45.83 (8.85)	46.00 (11.05)
Median (Q1, Q3)	47.0 (39.0, 53.0)	46.0 (38.6, 53.4)
^2^ Functional Reach Test [cm]:		
Mean (SD)	33.23 (11.09)	29.87 (9.29)
Median (Q1, Q3)	33.0 (25.5, 40.5)	30.0 (23.7, 36.3)
^2^ Timed Up and Go Test [s]:		
Mean (SD)	13.86 (9.61)	13.07 (6.56)
Median (Q1, Q3)	12.0 (6.5, 19.5)	12.0 (7.6, 18.4)
*^2^* 10 Meter Walk Test [m/s]:		
Mean (SD)	0.64 (0.21)	0.63 (0.25)
Median (Q1, Q3)	0.62 (0.50, 0.78)	0.62 (0.46, 0.80)
^2^ Falls Efficacy Scale—International [points]:		
Mean (SD)	32.59 (11.83)	28.47 (8.96)
Median (Q1, Q3)	32.0 (24.0, 40.8)	28.0 (22.0, 34.0)

Pts—patients; SD—standard deviation; Q1—first quartile, Q3—third quartile. ^1^ Chi-square test; ^2^ Mann–Whitney U test. The groups did not differ at baseline for any patients’ characteristics (*p* > 0.05).

**Table 3:** Pre-intervention characteristics of the groups, continued.

Characteristics	Experimental Group (n = 25)	Control Group (n = 25)
	Mean (SD) Median (Q1, Q3)	
**Assessment of postural balance on a stabilometric platform (60 s)**		
CoP path length [mm]	668.10 (378.70) 701.0 (412.57, 923.63)	622.45 (450.46) 688.41 (318.57, 926.33)
95% confidence ellipse area [mm^2^]	11.10 (6.27) 11.90 (6.87, 15.33)	10.50 (7.25) 10.12 (5.61, 15.39)
**Spatiotemporal gait parameters**		
Left side: step length [cm]	27.5 (8.9) 27.8 (21.50, 33.50)	27.6 (9.0) 27.9 (21.53, 33.67)
Left side: stance phase [%]	71.1 (4.5) 71.4 (68.06, 74.14)	69.7 (3.5) 69.94 (67.34, 72.06)
Right side: step length [cm]	27.4 (8.2) 27.95 (21.87, 32.93)	27.2 (8.1) 27.75 (21.74, 32.66)
Right side: stance phase [%]	69.0 (4.6) 69.31 (65.90, 72.10)	68.5 (4.0) 68.77 (65.80, 71.20)
Stride length [cm]	55.2 (15.9) 56.27 (44.48, 65.92)	55.7 (13.6) 56.62 (46.53, 64.87)
Stride time [s]	1.4 (0.24) 1.42 (1.24, 1.56)	1.5 (0.22) 1.52 (1.35, 1.65)
Step width [cm]	13.1 (3.9) 13.36 (10.47, 15.73)	12.2 (4.1) 12.48 (9.43, 14.97)
Double stance phase [%]	41.1 (8.5) 41.67 (35.37, 46.83)	40.3 (6.4) 40.73 (35.98, 44.62)
Cadence [step/min]	82.4 (14.7) 83.39 (72.48, 92.32)	80.9 (17.2) 82.06 (69.30, 92.50)
Velocity [km/h]	1.40 (0.4) 1.43 (1.13, 1.67)	1.30 (0.2) 1.31 (1.17, 1.43)

SD—standard deviation; Q1—first quartile, Q3—third quartile. The groups did not differ at baseline for any patients’ characteristics (Mann–Whitney U test *p* > 0.05).

**Table 4:** Within- and between-group comparison of functional tests’ results and fear of falling (n = 50).

Characteristics	Experimental Group (n = 25 ^1^)	Control Group (n = 25 ^1^)	^3^ Between-Group Level of Significance (*p*)
	Mean (SD) Median (Q1, Q3)		
**Berg Balance Scale [points]**			
Before	45.83 (8.85) * 44.84 (39.86, 51.80)	46.00 (11.05) * 45.67 (38.55, 53.45)	0.408
After	49.50 (7.82) * 49.10 (44.23, 54.77)	49.23 (9.80) * 49.22 (42.62, 55.84)	0.256
^2^ Within-group level of significance (*p*):	0.001	0.009	
**Functional Reach Test [cm]**			
Before	33.23 (11.09) 33.20 (25.76, 40.70)	29.87 (9.29) * 29.82 (23.61, 36.13)	0.150
After	34.47 (9.01) 34.41 (28.40, 40.54	34.50 (8.64) * 34.53 (28.68, 40.32)	0.870
^2^ Within-group level of significance (*p*):	0.513	0.021	
**Timed Up and Go Test [s]**			
Before	13.86 (9.61) * 13.84 (7.38, 20.34)	13.07 (6.56) * 13.04 (8.65, 17.49)	0.958
After	12.70 (7.49) * 12.67 (7.65, 17.75)	11.13 (4.88) * 11.11 (7.82, 14.44)	0.623
^2^ Within-group level of significance (*p*):	0.047	0.009	
**10 Meter Walk Test [s]**			
Before	0.64 (0.21) 0.62 (0.50, 0.78)	0.63 (0.25) * 0.60 (0.46, 0.80)	0.948
After	0.66 (0.23) 0.70 (0.55, 0.82)	0.70 (0.21) * 0.71 (0.56, 0.86)	0.543
^2^ Within-group level of significance (*p*):	0.170	0.015	
**Falls Efficacy Scale—International [points]**			
Before	32.59 (11.83) 32.56 (24.62, 40.56)	28.47 (8.96) * 28.45 (22.43, 34.51)	0.525
After	30.54 (11.83) 30.51 (22.57, 38.51)	26.10 (9.10) * 26.12 (19.96, 32.24)	0.527
^2^ Within-group level of significance (*p*):	0.160	0.002	

SD—standard deviation; Q1—first quartile, Q3—third quartile. SD—standard deviation; Q1—lower quartile; Q3—upper quartile; ^1^ In both the baseline and final outcome analyses, all randomized patients were included in accordance with the intention-to-treat analysis; ^2^ The Wilcoxon signed-rank test; ^3^ the Mann–Whitney U test; * statistically significant within-group differences between baseline and final study outcomes.

**Table 5:** Within- and between-group comparison of postural balance control assessments (60 s).

Characteristics	Experimental Group (n = 25 ^1^)	Control Group (n = 25 ^1^)	^3^ Between-Group Level of Significance (*p*)
	Mean (SD) Median (Q1, Q3)		
**CoP Path Length [mm]**			
Before	668.10 (378.70) 687.04 (412.80, 923.40)	622.45 (450.46) 599.93 (318.84, 926.06)	0.678
After	637.73 (400.82) 657.77 (367.58, 907.88)	585.80 (398.27) 565.89 (317.37, 854.23)	0.675
^2^ Within-group level of significance (*p*):	0.385	0.824	
**95% Confidence Ellipse Area [mm^2^]**			
Before	11.10 (6.27) 11.41 (6.87, 15.33)	10.50 (7.25) 10.14 (5.61, 15.39)	0.597
After	10.63 (6.60) 10.96 (6.18, 15.08)	9.77 (6.60) 9.44 (5.32, 14.22)	0.672
^2^ Within-group level of significance (*p*):	0.367	0.741	

CoP—center of pressure; SD—standard deviation; Q1—first quartile, Q3—third quartile. ^1^ In both the baseline and final outcome analyses, all randomized patients were included in accordance with the intention-to-treat analysis; ^2^ The Wilcoxon signed-rank test; ^3^ the Mann–Whitney U test.

**Table 6:** Within- and between-group comparison of gait analysis results.

Characteristics	Experimental Group (n = 25 ^1^)	Control Group (n = 25 ^1^)	^3^ Between-Group Level of Significance (*p*)
	Mean (SD) Median (Q1, Q3)		
**Left Side—Step Length [cm]**			
Before	27.5 (8.9) * 29.5 (23.5, 35.5)	27.6 (9.0) 26.0 (19.9, 32.1)	0.698
After	30.1 (9.8) * 28.0 (21.4, 34.6)	27.3 (7.4) 28.5 (23.5, 33.5)	0.750
^2^ Within-group level of significance:	0.045	0.736	
**Left Side—Stance Phase [%]:**			
Before	71.1 (4.5) 70.0 (66.0, 74.0)	69.7 (3.5) 71.0 (68.6, 73.4)	0.6735
After	68.0 (5.1) 69.5 (66.1, 72.9)	69.8 (3.3) 68.0 (65.8, 70.2)	0.4781
^2^ Within-group level of significance:	0.335	0.958	
**Right Side—Step Length [cm]:**			
Before	27.4 (8.2) * 25.0 (19.5, 30.5)	27.2 (8.1) 28.0 (22.5, 33.5)	0.9323
After	30.3 (9.0) * 32.0 (25.9, 38.1)	27.4 (8.4) 26.0 (20.3, 31.7)	0.3192
^2^ Within-group level of significance:	0.015	0.655	
**Right Side—Stance Phase [%]:**			
Before	69.0 (4.6) 70.5 (67.4, 73.6)	68.5 (4.0) 67.0 (64.3, 69.7)	0.4187
After	67.9 (5.0) 66.0 (62.6, 69.4)	68.4 (3.9) 69.0 (66.4, 71.6)	0.3630
^2^ Within-group level of significance:	0.954	0.708	
**Stride Length [cm]**			
Before	55.2 (15.9) 58.0 (47.3, 68.7)	55.7 (13.6) 53.0 (43.8, 62.2)	0.466
After	59.1 (16.8) 57.0 (45.7, 68.3)	55.1 (12.2) 56.0 (47.8, 64.2)	0.825
^2^ Within-group level of significance:	0.079	0.859	0.466
**Stride Time [s]**			
Before	1.4 (0.2) 1.5 (1.37, 1.63)	1.5 (0.2) 1.4 (1.27, 1.53)	0.610
After	1.52 (0.2) 1.4 (1.27, 1.53)	1.52 (0.2) 1.6 (1.47, 1.73)	0.955
^2^ Within-group level of significance:	0.816	0.653	
**Step Width [cm]**			
Before	13.1 (3.9) 12.0 (9.4, 14.6)	12.2 (4.1) 13.5 (10.7, 16.3)	0.057
After	13.8 (4.2) 15.0 (12.2, 17.8)	12.7 (4.3) 11.5 (8.6, 14.4)	0.302
^2^ Within-group level of significance:	0.444	0.150	
**Double Stance Phase [%]**			
Before	41.1 (8.5) 39.0 (33.3, 44.7)	40.3 (6.4) 42.0 (37.7, 46.3)	0.644
After	40.3 (7.9) 41.5 (36.2, 46.8)	41.1 (6.9) 39.0 (34.4, 43.6)	0.835
^2^ Within-group level of significance:	0.763	0.519	
**Cadence [step/min]**			
Before	82.4 (14.7) * 84.0 (74.1, 93.9)	80.9 (17.2) 79.0 (67.4, 90.6)	0.555
After	79.6 (17.0) * 77.0 (65.6, 88.4)	83.6 (18.1) 86.0 (73.8, 98.2)	0.250
^2^ Within-group level of significance:	0.029	0.354	
**Velocity [km/h]**			
Before	1.43 (0.36) 1.50 (1.26, 1.74)	1.31 (0.2) 1.25 (1.11, 1.39)	0.605
After	1.59 (0.39) 1.53 (1.27, 1.79)	1.36 (0.2) 1.40 (1.26, 1.54)	0.300
^2^ Within-group level of significance:	0.314	0.409	

SD—standard deviation; Q1—first quartile, Q3—third quartile. ^1^ In both the baseline and final outcome analyses, all randomized patients were included in accordance with the intention-to-treat analysis; ^2^ The Wilcoxon signed-rank test; ^3^ the Mann–Whitney U test; * statistically significant within-group differences between baseline and final study outcomes.

## Data Availability

The original contributions presented in this study are included in the article/Supplementary Material. Further inquiries can be directed to the corresponding author.

## Supplementary Materials

The following supporting information can be downloaded at: https://www.mdpi.com/article/10.3390/jcm14176142/s1, Table S1: Sensitivity analysis—rank-based ANCOVA (Quade/Conover–Iman).

## Abbreviations

The following abbreviations are used in this manuscript

UI	Uncertainty interval
RR	Relative risk
CI	Confidence interval
PBT	Perturbation-based balance training
TPBT	Treadmill perturbation-based balance training
RCT	Randomized clinical trial
EG	Experimental group
CG	Control group
ISRCTN	International Standard Randomized Controlled Trial Number
MMSE	Mini-Mental State Examination
GDS	Geriatric Depression Scale
BBS	Berg Balance Scale
FRT	Functional Reach Test
TUG	Timed Up and Go
ICC	Intraclass Correlation Coefficient
AUC	Area under the curve
10MWT	10-Meter Walk Test
FES-I	Falls Efficacy Scale–International
ITT	Intention-to-treat
SD	Standard deviation
Q1	Lower quartile
Q3	Upper quartile
CoP	Center of pressure
CoM	Center of mass
