# Supplementary material for: Assessment of Postural Control and Gait in Patients with Chronic Stroke After Treadmill Perturbation-Based Training: A Randomized Clinical Trial
Source: J Clin Med. 2025 Aug 30;14(17):6142. doi: 10.3390/jcm14176142 (PMC12429574; doi:10.3390/jcm14176142)
Supplement: Supplementary file 1 [file jcm-14-06142-s001.zip › jcm-3794987-supplementary.pdf]

Supplementary Table S1. Sensitivity analysis using rank-based ANCOVA (Quade/Conover–Iman). Post-intervention outcome adjusted for baseline; F-test for the group effect reported with η^2 and ω^2. Primary non-parametric analyses (Wilcoxon/Mann–Whitney; ITT) are presented in the main text.

Supplementary Table S1. Sensitivity analysis — rank-based ANCOVA (Quade/Conover–Iman).

<table>
<tr><th>Characteristic</th><th>Experimental group (n=25)</th><th>Control group (n=25)</th><th>Between-group level of significance (p)</th></tr>
<tr><td>Berg Balance Scale [points]</td><td colspan="2">Post (adjusted for baseline) — rank-ANCOVA (Quade/Conover–Iman): $F(1, 48) = 0.179$, $\eta^2 = 0.004$, $\omega^2 = 0.000$</td><td>0.674</td></tr>
<tr><td>Functional Reach Test [cm]</td><td colspan="2">Post (adjusted for baseline) — rank-ANCOVA (Quade/Conover–Iman): $F(1, 48) = 2.223$, $\eta^2 = 0.044$, $\omega^2 = 0.000$</td><td>0.143</td></tr>
<tr><td>Timed Up and Go Test [s]</td><td colspan="2">Post (adjusted for baseline) — rank-ANCOVA (Quade/Conover–Iman): $F(1, 48) = 0.352$, $\eta^2 = 0.007$, $\omega^2 = 0.000$</td><td>0.556</td></tr>
<tr><td>10 Meter Walk Test [s]</td><td colspan="2">Post (adjusted for baseline) — rank-ANCOVA (Quade/Conover–Iman): $F(1, 48) = 1.183$, $\eta^2 = 0.024$, $\omega^2 = 0.000$</td><td>0.282</td></tr>
</table>

Notes: Rank-based ANCOVA implemented as Quade/Conover–Iman procedure: ranks of post-intervention outcome regressed on ranks of baseline; residuals compared between groups via one-way ANOVA. Effect sizes are reported as η^2 and ω^2 derived from the ANOVA on residuals. Primary analyses in the manuscript used Wilcoxon (within-group) and Mann–Whitney (between-group changes) under ITT; the present sensitivity analysis yields concordant conclusions.
